# Dentist Empathic Accuracy Is Associated With Patient-Reported Reassurance

**DOI:** 10.1016/j.identj.2022.06.009

**Published:** 2022-07-25

**Authors:** Serge A. Steenen, Moniek A.J. Zeegers, Arjen J. van Wijk, Saif Al-Zubaidi, Minakshi Jethu-Ramkrishan, Aida Loddin, Jan de Lange, Ad de Jongh

**Affiliations:** a Department of Oral and Maxillofacial Surgery, Amsterdam University Medical Center, University of Amsterdam, Amsterdam, The Netherlands; b Department of Oral Public Health, Academic Centre for Dentistry Amsterdam, Amsterdam, The Netherlands; c Department of Psychology, Mondium GGz, Clinical Health Center, Beesd, The Netherlands; d Institute of Health and Society, University of Worcester, Worcester, UK; e School of Psychology, Queen's University, Belfast, Northern Ireland

**Keywords:** Dental anxiety, Cross-sectional studies, Dental staff, Empathic accuracy, Reassurance, Dental extractions

## Abstract

**Objectives:**

The aim of this work was to determine dentists’ ability to accurately estimate patients’ anxiety level during dental treatment (ie, “empathic accuracy”) and to determine the strength of the association between empathic accuracy and patient-reported reassurance.

**Methods:**

A cross-sectional study was conducted amongst 177 adult patients who underwent different invasive dental procedures (ie, extractions or procedures requiring injections and drilling) performed by 10 different dentists from 3 dental offices in the Netherlands. Patients reported their anxiety level during treatment and the extent to which they felt reassured by the dentist using a visual analogue scale (VAS). Simultaneously, the dentists estimated patients’ anxiety level. Empathic accuracy was calculated as an absolute difference between patient-reported anxiety (100-point VAS) and dentist estimation of anxiety (100-point VAS).

**Results:**

Agreement between dentists’ assessment of patients' anxiety and patient-reported anxiety proved good, intraclass correlation coefficient (177) = 0.63; 95% confidence interval [CI], 0.53-0.71. A small to medium-sized positive correlation, *r* (177) = 0.15; 95% CI, 0.00-0.29, was found between dentists’ empathic accuracy and patient-reported reassurance. A negative correlation was found between empathic accuracy and patients’ anxiety scores, *r* (177) = −0.23; 95% CI, −0.38 to −0.09.

**Conclusions:**

Given that greater empathic accuracy was associated with higher patient-reported reassurance during treatment, training young dental professionals in empathic accuracy might help patients feel reassured. Importantly, our results also suggest that with elevated levels of patient anxiety it is increasingly challenging for dentists to recognise this emotion, and thus support the patient in anoptimal manner.

## Introduction

A wide array of studies have established that dental anxiety is deeply rooted in previous negative dental experiences or, less frequently, in other types of distressing and traumatic life events.^1^, ^2^, ^3^, ^4^, ^5^, ^6^, ^7^, ^8^, ^9^ Risk factors for experiencing pathologic forms of dental anxiety (a spectrum ranging from elevated fear and dental phobia to posttraumatic stress disorder) include having undergone an invasive dental procedure (eg, injections, drillings, and extractions), extreme emotional responses during negative experiences (eg, felt helplessness, nausea, embarrassment, or sense of suffocation), and characteristics related to the dentist (eg, lack of understanding, impoliteness, rudeness, insufficient information provided during treatment, and criticism).^1^, ^2^, ^3^, ^4^, ^5^, ^6^^,^^10^, ^11^, ^12^ Creating a pleasant dental experience for the patient, and thereby preventing the development of a pathologic form of fear of the dental treatment, is most likely when the dentist is properly trained and willing to conduct behaviours that affect the internal state of the patient. In other words, it is imperative^13^, ^14^, ^15^, ^16^ that dentists have the empathic capabilities (we use the term “empathic accuracy”) to accurately estimate their patients’ level of anxiety and, moreover, to respond accordingly. Examples are providing emotional reassurance and a creating a sense of control by the patient.^12^^,^^17^^,^^18^

Regarding the accuracy with which dental professionals estimate their patients’ anxiety levels during treatment, only one cross-sectional study by Höglund et al. that included 1128 adult patients from several Swedish public dental offices has been conducted.^19^ The accuracy with which dental professionals estimated their patients’ anxiety levels was examined by obtaining dental anxiety scores before an annual oral checkup, whilst their dentist was asked to rate how anxious they thought the patients felt. Both ratings were found to be positively correlated (*r* = 0.45, *P* < .001), but because the authors found “no correlations between any of the clinicians’ and patients’ ratings of dental anxiety” (p. 458) within a small subgroup of highly anxious individuals, they concluded that “clinicians were unsuccessful in identifying a dentally anxious patient” (p. 455). An explanation for this finding might be that annual checkups are generally not invasive and do not rank amongst the most feared dental situations,^10^ and as such this may have led to relatively little variability in scores. However, empathic accuracy has not yet been studied during invasive dental procedures, and it is unknown to what extent empathic accuracy is related to the degree to which patients feel reassured during treatment.

Therefore, the first purpose of the present study was to determine dentists’ ability to accurately estimate patients’ anxiety level by conducting a cross-sectional study in 3 general dental clinics in the Netherlands, amongst patients undergoing dental treatments that have been shown to be perceived as invasive in previous studies.^3^^,^^10^^,^^11^ We assessed dentists’ accuracy in estimating their patients’ level of anxiety during treatment (ie, “empathic accuracy”) and the extent to which patients felt reassured during treatment. Second, based upon previous suggestions,^10^^,^^12^^,^^17^^,^^18^ we hypothesised that greater accuracy of dentists’ assessment of patients’ anxiety level (empathic accuracy) would be significantly associated with patients’ self-reported felt (perceived) reassurance during treatment. Third, because a previous study by Höglund et al.^19^ revealed that it proved more difficult to adequately estimate the anxiety level of highly anxious patients, we investigated whether this relationship could be replicated in our sample. Therefore, we also determined the association between dentists’ empathic accuracy and patients’ anxiety level. We hypothesised that dentists’ empathic accuracy would be significantly lower amongst patients reporting relatively high levels of anxiety than amongst patients reporting relatively low levels of anxiety. In the same vein, we determined the association between patients’ self-reported felt reassurance during treatment and patients’ anxiety levels. We hypothesised that felt reassurance would be significantly less amongst patients with relatively high anxiety levels vs patients with relatively low anxiety levels. We were also interested in patient-, dentist-, and treatment-related factors that may influence empathic accuracy. Specifically, we examined whether empathic accuracy would be associated with dentists’ years of experience, dentists’ and patients’ gender and age, and type of dental treatment.

## Methods

### Participants

Between December 2014 and April 2016, on 34 unscripted days selected based on the researchers’ availability, a total of 177 consecutive adult patients of 9 dentists in 3 general dental practices (adjacent to the residence of data collecting researchers) in The Netherlands (Amsterdam, the Hague, and Alphen aan den Rijn) who were scheduled for invasive dental treatments were included. At arrival in the waiting room for the treatment appointment, they were asked to participate in this study by a researcher (SAZ, MJR, or AL). After potential participants had given oral and written informed consent to the researcher, their dentists were informed about their participation. Patients were informed by the researcher that their dentist was blinded to their answers on study questionnaires. Demographic variables were subsequently recorded.

### Design

This study was designed as a cross-sectional observational study. The Institutional Review Board (IRB), “the Medical Ethics Committee of the Academic Medical Centre in Amsterdam, The Netherlands” decided that formal assessment by the IRB was not required for this observational study (decision W 15_084 # 15.0098). Participant data were anonymised and were stored on a protected server of the Academic Centre for Dentistry in Amsterdam, The Netherlands.

### Materials and procedure

All participants underwent an invasive dental treatment (ie, dental extractions or procedures requiring both injections and drilling; root canal treatments, crown and bridge preparations, and fillings).^3^^,^^10^^,^^11^ After leaving the treatment room, they were asked by a researcher to fill out a questionnaire in the Dutch language containing five 100-point Visual Analogue Scale (VAS) items^19^, ^20^, ^21^ ranging from 0 (“not/none at all”) to 100 (“extremely”). The first 2 VAS items pertained to the experience of the treatment that day (“To what extent did you feel reassured by the dentist during treatment?” and “How much anxiety did you experience, on average, during treatment?”). The patients were also requested to respond to 3 different VAS items pertaining to their experience of the treatment.

Separately, after treatment, the dentist, blinded to the patients VAS scores, was instructed by the researcher to imagine being that particular patient and to estimate the patient's level of anxiety during treatment (“How much anxiety did the patient express during treatment, according to you?”) using a 100-point VAS ranging from 0 (“none at all”) to 100 (“extremely much”). Finally, the dentist filled out a number of operative variables on the case report form (ie, duration of treatment, amount of local anaesthetic injected, and nature of the treatment).

### Empathic accuracy

Empathic accuracy was calculated as an absolute difference between patient-reported intraoperative anxiety (100-point VAS) and dentist estimation of the patients’ anxiety (100-point VAS). This means that an empathic accuracy of “0” represents perfect accuracy and that the maximum inaccuracy is “100” if the patient-reported anxiety is found to be 100 and the dentist estimates the anxiety to be 0, or vice versa. The direction of the difference is deliberately not addressed in these absolute values so that the group means are not averaged out by both positive and negative values and, as such, reflect the true (absolute) means. By means of this formula, we were able to quantify per data point (dentist estimated value vs patient-reported value) how accurately a dentist could infer the feelings from a patient and subsequently determine a correlation coefficient for these individual data points on a group level.

### Bias

To avoid the Hawthorne effect^22^ (ie, when people behave differently because they know they are being watched), patients were informed that their dentists were not aware of the answers they provided on their case report form.

### Sample size calculation

To ensure a sufficient sample size, a sample size calculation was conducted beforehand for the empathic accuracy (correlation coefficient) with G*Power 3.1 software.^23^ A power of 80% is seen as a standard in clinical studies.^24^ We calculated that, assuming a correlation of 0.45 between 2 measurements based on previous literature,^19^ with a power (1-β error probability) of 0.8 and an alpha (α error probability) set to 0.05, a total sample size of ≥36 participants would be required. However, because the aforementioned previous study was performed during annual oral checkups^19^ and found that higher patient anxiety scores were associated with lower empathic accuracy, we could not exclude the possibility that during the invasive dental procedures in our study, empathic accuracy would be found to be lower. Therefore, under the assumption of a small effect, an optimal sample size of ≥175 participants would be required.

### Statistical analysis

Degree of conformity between dentists and patients in rating the patient's anxiety was determined by calculating intraclass correlation coefficients (ICCs; measures of agreement), whereas the association between continuous variables was determined by calculating Pearson's product-moment correlation coefficients (linear association). For interpreting effect sizes in the present study, we used the recommendation of recent insights in psychology, considering Pearson's *r* correlations of 0.10, 0.20, 0.30, and 0.40 to be small, typical, large, and very large, respectively.^25^^,^^26^ If distributions of continuous variables were skewed (felt reassurance), variables were (natural log) transformed. In addition to the main analyses, we tested the effects of dentist-, patient-, and treatment-related factors (eg, gender and years of dentist's experience) on patients’ ratings concerning anxiety and empathic accuracy. Paired *t* tests were used to compare the means of 2 dependent groups (dentists and patients). One-way analysis of variance was used to compare means of more than 2 unpaired groups (patients, grouped by treatments). Subgroup analyses were performed if explanatory variables contained outliers and, based on previous literature,^19^ a median split analysis^27^ was performed to explore empathic accuracy in a subgroup of highly anxious patients. All the statistical analyses were carried out using SPSS Statistics for Macintosh, Version 25.0 (IBM Corp.). The level of significance for all statistical analyses in this study was set at *a =* 0.05 (2-tailed). We did not adjust for multiple testing, because correcting for capitalisation on chance is based on the assumption that the null hypothesis is always true but a reference value for the main outcome (empathic accuracy) has been previously reported; hence, correcting for multiple testing in order to reduce the type I error is of less importance than reducing the chance of type II errors.^28^

## Results

A total of 177 consecutive patients were included. Initially, 189 individuals were asked to participate; 12 refused to take part in this study for personal reasons. The mean age was 41.4 years (range, 19-90). Ten dentists took part in this study, with a mean of 6.1 years of professional experience (range, 1-31). Descriptive statistics are displayed in Table 1, and the strengths of all associations are displayed in a Pearson correlation matrix (Table 2). The data that support the findings of this study are available from the corresponding author upon reasonable request.

**Table 1:** Descriptive statistics.

			M	SD
Patient-reported intraoperative anxiety	Overall (N = 177)		41.1	27.9
	Patients grouped by patient gender	Female (n = 80)	52.3	28.1
		Male (n = 97)	31.0	27.3
	Patients grouped by treatment	Dental extractions (n = 38)	54.5	20.7
		Crown/bridge preparations (n = 23)	49.1	24.4
		Root canal treatment (n = 45)	36.4	27.3
		Filling (n = 70)	33.6	29.4
	Patients grouped by dentist gender	Male dentist (n = 134)	42.3	27.5
		Female dentist (n = 43)	37.6	29.0
Dentist-estimated intraoperative anxiety	Overall		39.0	27.3
	Dentists grouped by gender	Male dentist (n = 134)	38.0	24.7
		Female dentist (n = 43)	39.4	28.1
Absolute difference between patient-reported intraoperative anxiety and dentist estimation	Overall (N = 177)		16.9	16.7
	Dentists grouped by gender	Male dentist (n = 134)	16.3	15.5
		Female dentist (n = 43)	18.5	20.1
Patient-reported intraoperative reassurance	Overall (N = 177)		80.7	20.6
	Dentists grouped by gender	Male dentist (n = 134)	77.9	20.6
		Female dentist (n = 43)	89.2	18.5

**Table 2:** Pearson correlation matrix.

	Patient age	Dentist experience (years)	Intraoperative anxiety	Empathic accuracy	Perceived reassurance
Patient age	1.000	0.247	0.053	0.048	0.189
Dentist experience (years)	0.247	1.000	0.138	0.068	0.030
Intraoperative anxiety	0.053	0.138	1.000	0.230	0.232
Empathic accuracy	0.048	0.068	0.230	1.000	0.151
Perceived reassurance	0.189	0.030	0.232	0.151	1.000

### Empathic accuracy

The mean self-reported intraoperative anxiety level as reported by the patients did not differ from the mean intraoperative patient anxiety level as estimated by the dentists, *t* (176) = −1.19; 95% confidence interval [CI], −1.39 to 5.63; *P* = .24. The ICC (agreement) and Pearson's product moment (association) were determined between the actual patient-reported intraoperative anxiety and the dentist's estimation of their patients’ anxiety level, ICC (177) = 0.63; 95% CI, 0.53-0.71; *r* (177) = 0.63; 95% CI, 0.53-0.71, *P* < .001.

### Empathic accuracy in relation to patients’ intraoperative anxiety

A negative correlation was found between empathic accuracy and patient-reported intraoperatively anxiety, *r* (177) = −0.23; 95% CI, −0.38 to −0.09; *P* = .002; that is, the more anxiety the patients experienced, the worse the dentists were able to adequately estimate their patients’ anxiety level (Fig.). A median split analysis showed that in the subgroup of patients with a score of ≥40 (n = 95), the empathic accuracy was significantly lower than in the subgroup of patients with a score of <40 (n = 82; M = 13.96 vs 19.37, respectively [28% lower], *t* [175] = 2.17; 95% CI, 0.49-10.32; *P* = .03). In the subgroup of patients with a score of ≥40 (ICC [95] = 0.381; 95% CI, 0.20-0.61), the ICC of the empathic accuracy was significantly lower than in the subgroup of patients with a score of <40 (ICC [82] = 0.394; 95% CI, 0.20-0.64). The fourth quartile VAS score for patients’ intraoperative anxiety was found to be 60/100, and only 4% of patients reported a VAS score ≥90. In the subgroup of patients with a score ≥60, the empathic accuracy was significantly lower than in the subgroup of patients with a score of <60 (M = 14.00 vs 20.81, respectively; 32% lower), *t* (175) = 2.33; 95% CI, 0.94-11.20; *P* = .02. In the subgroup of patients with a score ≥90, the empathic accuracy was significantly lower than in the subgroup of patients with a score <90 (M = 16.10 vs 36.43, respectively; 56% lower), *t* (175) = 3.25, 95% CI, 8.00-32.75, *P* < .001.

**Figure:**
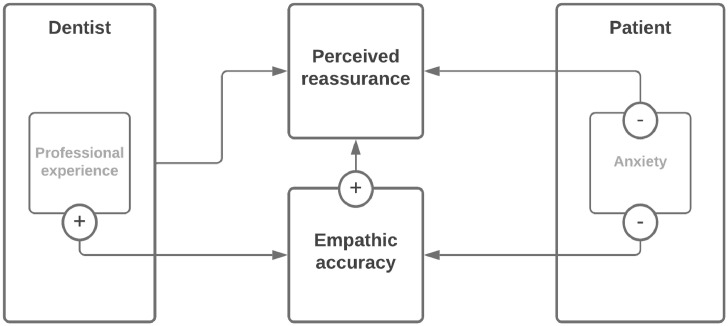
Directed acyclic graph showing relations between recorded variables in this study on dentist “empathic accuracy” and patient reassurance. With increasing years of professional experience, dentists were better at estimating their patients’ anxiety level. The more anxiety the patients experienced, the worse the dentists were able to adequately estimate their patients’ anxiety level. The more empathically accurate the dentist was, the more likely it was that patients reported higher levels of reassurance by their dentist. The more anxiety the patients experienced, the worse the dentists were able to adequately reassure their patients.

### Empathic accuracy in relation to felt reassurance

A positive correlation was found between empathic accuracy and patient-reported intraoperatively felt reassurance, *r* (177) = 0.15; 95% CI, 0.00-0.29; *P* < .05; that is, the more empathically accurate the dentist was, the more likely it was that patients reported higher levels of reassurance by their dentist (Fig.). Analyses with (natural log) transformed variables yielded similar results, *r* (177) = 0.21; 95% CI, 0.06-0.34; *P* = .006.

### Felt reassurance in relation to patients’ anxiety level

A negative correlation was found between felt reassurance and patient-reported intraoperatively perceived anxiety scores, *r* (177) = −0.23; 95% CI, −0.39 to −0.09; *P* = .002; that is, the more anxiety the patients experienced, the worse the dentists were able to adequately reassure their patients (Fig.). A median split analysis showed that in the subgroup of patients with a score ≥40 (n = 95), felt reassurance scores were significantly lower than in the subgroup of patients with a score <40 (n = 82; M = 76.1 vs 86.0, respectively), *t* (175) = 3.30; 95% CI, 3.96-15.88; *P* = .001.

### Felt reassurance in relation to dentist factors

Multiple linear regression analyses to predict perceived reassurance based on empathic accuracy and dentists’ years of professional experience yielded no significant regression equation, *F*(2, 174) = 2.07; *P* = .13), with an *r*^2^ of 0.02. Regarding dentist gender, patients felt statistically significantly better reassured during treatment when treated by female dentists than by male dentists, *t* (175) = 3.21; 95% CI, 4.34-18.22; *P* = .002.

### Factors related to empathic accuracy

*Dentist-related factors.* No statistically significant difference was found between male and female dentists in mean empathic accuracy, *t* (175) = −0.73; 95% CI, −7.93 to 3.64; *P* = .47. Likewise, no significant correlation between dentists’ years of professional experience and the empathic accuracy was found. However, after removing the data of one dentist with 31 years of experience (who showed to be a statistical outlier; *z* score > 3.29^29^), the results showed a medium-sized positive correlation between empathic accuracy and the dentists’ years of experience, *r* (174) = 0.21; 95% CI, 0.19-0.46; *P* = .006 (Fig.).

*Patient-related factors.* No statistically significant difference in dentists’ mean empathic accuracy was found between male and female patients, *t* (175) = 0.942; 95% CI, −2.60 to 7.36; *P* = .35. Likewise, no significant correlation between patients’ age and empathic accuracy was found.

*Treatment-related factors.* The mean values of empathic accuracy for the different treatments did not differ from each other, *F*(5, 171) = 1.27; 95% CI, −1.22 to 3.77; *P* = .28.

## Discussion

This is the first study to examine the empathic accuracy of dentists during invasive dental treatment. Patients’ self-reported intraoperative anxiety level did not statistically significantly differ from dentists’ estimation of patients’ intraoperative anxiety level, and the effect size for empathic accuracy was found to be very large. Whereas in general dentists’ empathic accuracy and reassurance felt by patients were found to be high, these were found to be lower amongst highly anxious patients. Also, the results were supportive of our hypothesis in that greater accuracy of dentists’ assessment of patients’ anxiety level was found to be associated with greater felt reassurance during treatment, by a typical effect size. This suggests that, generally, dentists are capable of estimating patients’ anxiety level during invasive treatments but that the more anxiety patients experience, the less dentists are capable of adequately estimating their patients’ anxiety level and reassuring their patients. Further, empathic accuracy was not found to be associated with dentists’ years of experience, dentists’ and patients’ gender, patients’ age, or type of dental treatment.

We found support for our hypothesis that increasing intraoperative anxiety levels would be associated with decreasing empathic accuracy, a relation previously found by Höglund et al.^19^ Likewise, we found support for our hypothesis that when people experience high levels of anxiety, dentists are less capable of reassuring their patients. Both results may possibly be explained by a phenomenon termed “dissociation,”^30^ a state of mind that occurs when threat and tension become too overwhelming and in which certain thoughts, emotions, perceptions, or disturbing memories are placed outside of consciousness. If this type of unresponsiveness to external stimulation would occur in highly anxious dental patients, it may cause inhibited expression of anxious behaviour, potentially leading to a faulty underestimation by a dentist of the patient's true level of anxiety. The logical consequence of this is that they are also less capable of offering their patients the reassurance they might need.

Regarding potential demographic factors that influence empathic accuracy (Fig.), the results of the study by Höglund et al.^19^ showed that older dentists and older patients were associated with greater empathic accuracy. In contrast to their findings, the present study showed no significant associations between either dentists’ years of professional experience or patient age and their empathic accuracy. However, after excluding one outlier, the results did show a medium-sized positive correlation between dentists’ empathic accuracy and their years of experience. These findings may be explained either by how familiar the older dentists were with their patients due to a longer treatment relationship or by increased experience in treating different types of patients in general.

Regarding factors that influence felt reassurance, the results of the present study also showed that patients felt significantly more reassured during treatment when treated by female dentists, compared to male dentists, by a medium-sized effect size (Cohen's *d* = 0.57). This may indicate that feminine characteristics may be effective characteristics of a reassuring practitioner.

Strengths of our study design include the context of highly feared (invasive) nature of dental treatments, allowing for higher levels and greater variability of patient's intraoperative anxiety. Although it is principally challenging to generalise findings to other samples in other countries, the present sample consisted of 10 different dentists with variable years of professional experience, with data from 3 different dental offices. With this, we hope to have supported not only the internal validity but also the external validity of the findings. A stark limitation of the present study, however, is that it had a cross-sectional design rendering it impossible to demonstrate causality between the variables we examined. Furthermore, socioenvironmental factors may contribute to empathic accuracy and one's ability to feel reassured by one's care provider. For example, an individuals’ ability to attend or utilise oral health care is, in part, due to social determinants such as race/ethnicity, socioeconomic status, and rurality.^8^ These variables were not recorded in this study, and we encourage future studies to further elaborate on this important aspect. Another limitation included the VAS, which has not yet been validated in Dutch-speaking patients and dentists. Furthermore, future research may benefit from acute treatment setups including patients with dental anxiety who avoid treatment.

To our knowledge, the extent to which patients feel reassured during dental treatment has not previously been evaluated. To this end, our finding suggests that better empathic accuracy may result in higher levels of felt reassurance. The act of reassuring patients has been common practice for decades in specialised dental fear clinics, in which behavioural management include simple reassurance besides “tell-show-do” techniques, gradual exposure, and relaxation exercises, an approach that has been found to be effective in reducing dental anxiety severity until 1 year after treatment.^31^ Given that greater empathic accuracy was found to be associated with higher patient-reported reassurance during treatment, training young dental professionals in empathic accuracy could help patients to feel reassured and supported, for example, using direct feedback to increase the learning curve of their empathic accuracy by means of comparing patient-reported and dentist-estimated anxiety with VAS. Importantly, our results suggest that with elevated levels of patient anxiety it becomes increasingly challenging for dentists to recognise anxiety and thus to support the patient in an adequate way. As it has been previously found that acknowledging the patient's dental anxiety in the first 2 minutes of the consultation reduces anxiety at 3-month follow-up, it may be advised to dental practitioners to routinely use a VAS assessing patients’ intraoperative anxiety levels.^32^

## Author contributions

**Conceptualisation:** Ad de Jongh, Arjen van Wijk. **Methodology:** Serge Steenen, Arjen van Wijk, Ad de Jongh. **Formal analysis:** Serge Steenen, Moniek Zeegers, Arjen van Wijk, Saif Al-Zubaidi S, Aida Loddin, Minakshi Jethu-Ramkrishan. **Investigation:** Saif Al-Zubaidi S, Aida Loddin, Minakshi Jethu-Ramkrishan. **Writing – original draft preparation:** Serge Steenen, Ad de Jongh; Moniek Zeegers, Arjen van Wijk, Saif Al-Zubaidi S, Aida Loddin, Minakshi Jethu-Ramkrishan, Jan de Lange. **Writing – review and editing:** Serge Steenen, Moniek Zeegers, Jan de Lange, Ad de Jongh. **Supervision:** Ad de Jongh, Serge Steenen, Arjen van Wijk, Jan de Lange. **Project administration:** Ad de Jongh, Serge Steenen.

## Conflict of interest

None disclosed.

## Appendix Supplementary materials

Image, application 1

Image, application 2

Image, application 3
