# Supplementary material for: Dentist Empathic Accuracy Is Associated With Patient-Reported Reassurance
Source: Int Dent J. 2022 Jul 25;73(1):101–7. doi: 10.1016/j.identj.2022.06.009 (PMC9875228; doi:10.1016/j.identj.2022.06.009)
Supplement: Supplementary file 1 [file mmc1.doc]

**To be completed by the dentist**

What was the duration of the treatment:

| □ <1 minute | □ 1 – 5 mins | □ 6 - 15 mins | □ 16 - 30 mins | □ > 30 mins |
| --- | --- | --- | --- | --- |
| Was there a difficult/complicated procedure?  □ Yes, because:………………………….  □ No | | | | |

The treatment included (tick the appropriate box):

| □ Anesthetic injections in the upper jaw, number of carpules: |
| --- |
| □ Anesthetic injections in the lower jaw, number of carpules: |
| □ Extraction …………… dental element(s) number(s)….  □ Surgical removal …………… dental element(s) number(s)….  □ Root canal treatment …………… dental element(s) number(s)….  □ Crown/bridge preparation …………… dental element(s) number(s)…. |

Something else: …

Would you please indicate on a scale from 0 to 10 how anxious you think the patient was during treatment. “0” stands for no anxiety at all. “10” stands for extremely anxious.


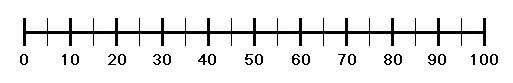


0 = No anxiety at all 100 = Extremely anxious

**For the patient**

***The first 2 questions are about what you experienced during the treatment.***

1. To what extent did you feel reassured by the dentist during the treatment?


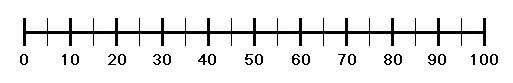


0 = Not at all reassured 100 = Extremely reassured

2. On average, how anxious did you feel during the treatment?


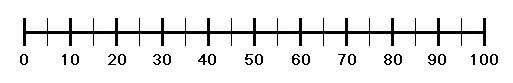


0 = No anxiety at all 100 = Extremely anxious

***These next 3 questions are about what you are experiencing right now.***

3. How satisfied are you with the treatment you just had?


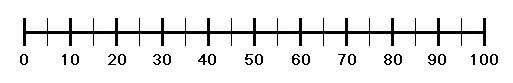


0 = Not at all satisfied 100 = Extremely satisfied

4. How emotionally charged is the memory of the treatment you just had, when you think about it now?


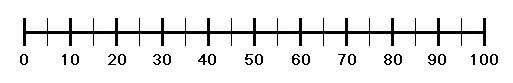


0 = No emotional charge 100 = Extremely emotionally charged

5. How vivid is the memory of the treatment you just had, when you think about it now?


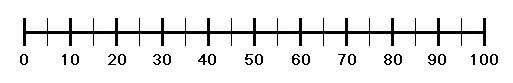


0 = Not at all vivid 100 = Extremely vivid
