# Supplementary material for: Dentist Empathic Accuracy Is Associated With Patient-Reported Reassurance
Source: Int Dent J. 2022 Jul 25;73(1):101–7. doi: 10.1016/j.identj.2022.06.009 (PMC9875228; doi:10.1016/j.identj.2022.06.009)
Supplement: Supplementary file 2 [file mmc2.doc]

**Patientnummer: ……
 Datum: ………………**

**VERRICHTINGENFORMULIER**

**Instructie (in te vullen door de tandarts)**

In het kader van dit onderzoek willen wij u vriendelijk verzoeken kort deze vragen in te vullen.

Duur van de **zojuist uitgevoerde** behandeling:

| □ <1 minuut | □ 1 – 5 min | □ 6 - 15 min | □ 16 - 30 min | □ > 30 min |
| --- | --- | --- | --- | --- |
| Was er sprake van een lastige / gecompliceerde procedure?  □ Ja, nl.:………………………………………………………….  □ Nee | | | | |

De **zojuist uitgevoerde** behandeling hield het volgende in (kruis aan wat u heeft gedaan):

| □ Verdoving in de bovenkaak, aantal carpules: |
| --- |
| □ Verdoving in de onderkaak, aantal carpules: |
| □ Extractie …………… gebitselement(en), elementnummer(s)….  □ Chirurgische behandeling …………… gebitselement(en), elementnummer(s)….  □ Wortelkanaalbehandeling …………… gebitselement(en), elementnummer(s)….  □ Kroon/brug preparatie …………… gebitselement(en), elementnummer(s)…. |

Iets anders, namelijk:

…………………………………………………………………………………………………………………

Wilt u alstublieft d.m.v. een cijfer van 0 tot 10 aangeven hoeveel angst de patiënt **volgens u** tijdens de behandeling vertoonde. “0” staat voor helemaal geen angst. “10” staat voor extreme angst.

| 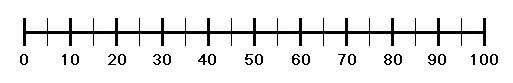 | |
| --- | --- |
| 0 = Helemaal geen angst | 100 = Extreem veel angst |

**Patientnummer: ……
 Datum: ………………**

**Visual Analogue Scales**

**De eerste 2 vragen gaan over wat u ervoer tijdens de behandeling.**

1. In hoeverre voelde u zich *tijdens de behandeling* gerustgesteld door de tandarts?

| 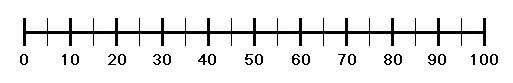 | |
| --- | --- |
| 0 = Helemaal niet gerustgesteld | 100 = Ontzettend gerustgesteld |

2. Hoeveel angst ervoer u gemiddeld *tijdens de behandeling*?

| 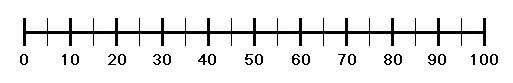 | |
| --- | --- |
| 0 = Helemaal geen angst | 100 = Extreem veel angst |

**Deze volgende 3 vragen gaan over wat u nu ervaart.**

3. Hoe *tevreden* bent u over de behandeling?

| 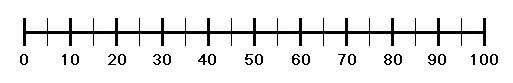 | |
| --- | --- |
| 0 = Helemaal niet tevreden | 100 = Extreem tevreden |

4. Hoe *naar* is de herrinering als u er nu aan terugdenkt?

| 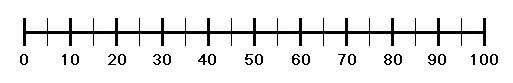 | |
| --- | --- |
| 0 = Helemaal niet naar | 100 = Extreem naar |

5. Hoe *levendig* is de herinnering als u er nu aan terugdenkt?

| 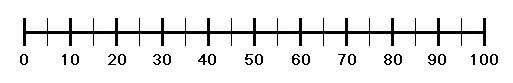 | |
| --- | --- |
| 0 = Helemaal niet levendig | 100 = Extreem levendig |
